# Clinical usefulness of lipid ratios, visceral adiposity indicators, and the triglycerides and glucose index as risk markers of insulin resistance

**DOI:** 10.1186/s12933-014-0146-3

**Published:** 2014-10-20

**Authors:** Tingting Du, Gang Yuan, Muxun Zhang, Xinrong Zhou, Xingxing Sun, Xuefeng Yu

**Affiliations:** Department of Endocrinology, Tongji Hospital, Tongji Medical College, Huazhong University of Science and Technology, Wuhan, 430030 China; Department of Anesthesiology, Tongji Hospital, Tongji Medical College, Huazhong University of Science and Technology, Wuhan, 430030 China

**Keywords:** Insulin resistance, Apolipoprotein B, Apolipoprotein A-I, Visceral adiposity index, Lipid accumulation product, TyG index

## Abstract

**Background:**

To directly compare traditional lipid ratios (total cholesterol [TC]/high density lipoprotein cholesterol [HDL-C], non-HDL-C/HDL-C, low density lipoprotein cholesterol [LDL-C]/HDL-C, and triglycerides [TG]/HDL-C), apolipoprotein B (apoB)/apolipoprotein A-I (apoA-I) ratio, visceral adiposity index (VAI), lipid accumulation product (LAP), and the product of TG and fasting glucose (TyG) for strength and independence as risk factors for insulin resistance (IR).

**Methods:**

We conducted a cross-sectional analysis of 7629 Chinese adults using data from the China Health and Nutrition Survey 2009.

**Results:**

For all lipid ratios (traditional lipid ratios and apoB/apoA-I), among both sexes, TG/HDL-C explained the most additional percentage of variation in HOMA-IR (2.9% in men, and 2.3% in women); for all variables of interest, the variability in HOMA-IR explained by VAI and TG/HDL-C were comparable; TyG had the most significant association with HOMA-IR, which explained 9.1% for men and 7.8% for women of the variability in HOMA-IR. Logistic regression analysis showed the similar patterns. Receiver operating characteristic (ROC) curve analysis showed that, among both sexes, TG/HDL-C was a better discriminator of IR than apoB/apoA-I; the area under the ROC curve (AUC) for VAI (0.695 in men and 0.682 in women) was greater than that for TG/HDL-C (AUC 0.665 in men and 0.664 in women); TyG presented the greatest value of AUC (0.709 in men and 0.711 in women).

**Conclusion:**

The apoB/apoA-I performs no better than any of the traditional lipid ratios in correlating with IR. The TG/HDL-C, VAI and TyG are better markers for early identification of IR individuals.

## Background

Cardiovascular disease (CVD) is now the leading cause of morbidity and mortality worldwide. The new epidemic of diabetes is fuelling an alarming increase in premature CVD incidence. Insulin resistance (IR) and the consequence of compensatory hyperinsulinemia are fundamentally pathogenetic factors for a set of metabolic abnormalities [1], which contribute to the development of diabetes and CVD. Thus, detection and treatment of IR before the manifestation of clinical disease are of paramount importance. Although hyperinsulinemic-euglycemic clamp is the gold standard test for measurement of IR, it is impractical in clinical settings due to practical, ethical and economic reasons. Therefore, markers of IR which are simple and easy to implement are urgently needed. Dyslipidemia is a major risk factor for CVD. There is evidence suggesting that traditional lipid ratios, such as total cholesterol (TC)⁄ high density lipoprotein cholesterol (HDL-C), non-HDL-C/HDL-C, and triglycerides (TG)/HDL-C, which take account of the proportion between the pro-atherogenic and anti-antherogenic fractions, are more effective than single measures of lipids in detecting IR [2]. A plethora of studies have shown that apolipoprotein B (apoB)/apolipoprotein A1 (apoA-I) (in which apo B means apo B100) ratio is superior to traditional lipids in CVD prediction [3-5]. Moreover, one study indicates that apoB/apoA-I is significantly associated with IR [6]. These analyses have fuelled the recommendation that apoB and apoA-I should be measured in routine clinical care [7]. It is uncertain, however, whether the apoB/apoA-I ratio is superior to traditional lipid ratios in detecting IR. Though head-to head comparisons between apoB/apoA-I and traditional lipid ratios have been conducted to evaluate whether apoB/apoA-I could be used instead of traditional lipid ratios for CVD risk [8], direct comparisons between these measurements to identify IR are limited.

On the other hand, emerging evidence indicates that visceral adiposity is associated with IR [9,10]. The visceral adiposity index (VAI), a mathematical model that uses both anthropometric (body mass index [BMI] and waist circumference [WC]) and metabolic (TG and HDL-C) parameters, and lipid accumulation product (LAP), a mathematical model that based on a combination of TG and WC, are sensitive markers of visceral obesity [11,12], and have the ability to identify IR. Since both the VAI and LAP incorporates TG along with indicators of adiposity (WC), they might be superior markers of IR compared with lipid ratios, as regional fat distribution can modulate cardiometabolic risk [9,13]. It has also been suggested that the product of TG and fasting glucose (TyG) has high sensitivity for recognizing IR [14,15]. Since TyG incorporates TG along with fasting plasma glucose (FPG), superiority of TyG in identifying IR might be achieved as both TG and FPG are well validated for their roles in IR [16,17]. To the best of our knowledge, limited data are available regarding direct comparisons between the variables mentioned above in identifying IR.

Hence, the aims of this study were to: 1) evaluate the performance of a range of lipids and apolipoproteins, as well as relevant ratios in identifying IR; 2) directly compare the traditional lipid ratios, apoB/apoA-I ratio, VAI, LAP, and TyG for strength and independence as risk factors for IR.

## Methods

### Study population

We used data from the China Health and Nutrition Survey (CHNS) for our analysis. The CHNS is the only large-scale longitudinal, household-based survey in China. Full details of the study have been described elsewhere [18]. Briefly, the CHNS rounds were conducted in 1989, 1991, 1993, 1997, 2000, 2004, 2006, and 2009. For each round, a stratified multistage, random cluster process was employed to draw study sample from each of the nine provinces (Liaoning, Heilongjiang, Jiangsu, Shandong, Henan, Hubei, Hunan, Guangxi, and Guizhou), covering approximately 56% of China’s population, that vary significantly in terms of geography, economic development, and health status. Each participant provided a written informed consent and the study was approved by the institutional review committees of the University of North Carolina at Chapel Hill, the National Institute of Nutrition and Food Safety, Chinese Center for Disease Control and Prevention, and the China-Japan Friendship Hospital, Ministry of Health.

Since fasting blood samples were initially collected in 2009, this study examined data from CHNS 2009. All participants were asked to complete a structured questionnaire which provided information on educational attainment, cigarette smoking and alcohol consumption habits, histories of current and previous illness, and medical treatment. A total of 10,038 adult respondents were surveyed at the 2009 exam, 1,423 did not give blood, 402 were not fasting before blood collection, and 62 were pregnant, resulting in a total of 8,151 individuals with fasting blood samples. Exclusion criteria included a self-reported diabetes diagnosis or diabetes medication use, chronic kidney disease (estimated glomerular filtration rate <15 ml/min per 1.73 m^2^), and extreme BMI (≥40 kg/m^2^), TG (>500 mg/dl) or HDL-C (>100 mg/dl) values. The remaining 7629 participants with anthropometry and clinical examination information were included in current analysis.

### Measurements

Weight, height, WC and blood pressure (BP) were measured following standardized protocols from the World Health Organization. Weight was measured with participants wearing light clothing on a calibrated beam scale and height was measured without shoes using a portable SECA stadiometer. BMI was calculated as weight (in kilograms) divided by the square of height (in meters). WC was measured with an inelastic tape at a midpoint between the bottom of the rib cage and the top of the iliac crest at the end of exhalation. Seated systolic/diastolic BP was measured by trained technicians in triplicate after a 10-min rest, using mercury manometers. The three readings were averaged.

### Biochemical measurements

Blood was collected after an at least 8-hour overnight fast. All samples were analyzed in a national central lab in Beijing, with strict quality control. FPG was measured by the GOD-PAP method (Randox Laboratories Ltd, UK]. All lipids (TC, TG, low density lipoprotein cholesterol [LDL-C], and HDL-C) were directly measured with Hitachi 7600 automated analyzer (Hitachi Inc., Tokyo, Japan). TC, LDL-C, and HDL-C were measured enzymatically (Kyowa, Japan). Non-HDL-C was calculated as TC minus HDL-C. TG was measured by GPO-PAP method (Kyowa, Japan). ApoA-I and apoB were measured by immunoturbidimetric method (Randox Laboratories Ltd, UK). Hypersensitive C-reactive protein (hs-CRP) was determined by immunoturbidimetric method (Denka Seiken,Japan reagents). Fasting insulin concentration was measured using the radioimmunology assay (Gamma counter XH-6020, Beijing, China). Homeostasis model assessment of insulin resistance (HOMA-IR) was calculated by the formula: HOMA-IR = fasting insulin (micro-international units per milliliter) × FPG (millimoles per liter)/22.5.

LAP, VAI, and TyG were calculated using the published formula. LAP [12]: Men: [WC (cm) – 65] × [TG (mmol/l)]; Women: [WC (cm) – 58] × [TG (mmol/l)]. To avoid having nonpositive values for LAP, we reassigned any WC values for men/women that were less than 65/58 cm to 66.0/59.0 cm. VAI [11]: Men: [WC/39.68 + (1.88 × BMI)] × (TG/1.03) × (1.31/HDL); Women: [WC/36.58 + (1.89 × BMI)] × (TG/0.81) × (1.52/HDL), where both TG and HDL levels are expressed in mmol/l. The TyG index [14,15]: Ln [TG (mg/dl) × FPG (mg/dl)/2].

### Definitions

According to the International Diabetes Federation recommendations for Asians [19], central obesity is defined as WC ≥90 cm for men and ≥80 cm for women.

Quartiles of the HOMA-IR were calculated separately for men and women with FPG <126 mg/dl. IR was defined as having HOMA-IR in the upper quartile of the HOMA index.

The Framingham risk score (FRS) was calculated according to the National Cholesterol Education Program-Adult Treatment Panel III algorithm [20]. According to FRS, individuals were categorized into 3 risk groups (low [<10%], intermediate [10–20%], and high [≥20%]).

### Statistical analysis

All statistical analyses were conducted using SPSS software (version 12.0 for windows; SPSS, Chicago, IL, USA). Continuous variables were expressed as medians and interquartile ranges (IQR) due to their skewed distribution and were compared with Mann-Whitney U test between participants with and without IR. Categorical variables were presented as percentages and were compared with a Chi-square test. Variables of interest were TC, TG, LDL-C, HDL-C, apoB, apoA-I, TC/HDL-C, TG/HDL-C, LDL-C/HDL-C, non-HDL-C/HDL-C, apoB/apoA-I, LAP, VAI, and TyG. Before conducting the linear regression analyses, all these variables except TyG were log-transformed (natural logarithm) to approximate normal distributions. Multiple linear regression analysis with log HOMA-IR as the dependent variable was used to assess associations of these log-transformed variables as well as TyG with HOMA-IR. The predictive values of each variable were judged by comparing the proportion of the total variation that each of the indexes could explain, that is, the R^2^ for the entire regression model minus the R^2^ for a base model that excluded each of the indexes. For each index, we divided them into increasing sex-specific quartile values and used logistic regression analysis to calculate odds ratios (ORs) and 95% confidence intervals (CIs) for IR comparing those in quartiles 2 through 4 with those in quartile 1. Three models were fitted. Model 1 was adjusted for age, socioeconomic status (rural/urban settings, region, and education level), smoking status, alcohol use, BMI, systolic BP, uric acid, and hs-CRP. Model 2 was additionally adjusted for WC. Model 3 was adjusted for BMI, socioeconomic status, alcohol use, and FRS. A receiver operating characteristic (ROC) curve analysis was performed for each measure to compare the abilities of these measures to correctly discriminate IR. The overall diagnostic accuracy was quantified using the area under the ROC curve (AUC). Significance was accepted at a two-tailed P <0.05.

## Results

Insulin resistant individuals were more likely than insulin sensitive counterparts to be older. BMI, WC, systolic and diastolic BP, TC, TG, LDL-C, non-HDL-C, apoB, TC/HDL-C, TG/HDL-C, LDL-C/HDL-C, non-HDL-C/HDL-C, apoB/apoA-I, LAP, VAI, TyG, FPG, HOMA-IR, uric acid, and hs-CRP were significantly higher, whereas HDL-C and apoA-I were lower in insulin resistant individuals than in insulin sensitive counterparts (Table 1). There were significant differences in lipid and apolipoprotein profiles between men and women; therefore all following analyses were stratified by sex.

**Table 1:** **Characteristics of the study participants according to gender or presence of insulin resistance (IR)**

	**Men**	**Women**	**P (Men vs women)**	**Non-IR**	**IR**	**p (IR vs non-IR)**
n	3619	4010		4829	2800	
Age, year	50.6 (39.3-60.7)	51.0 (40.3-60.6)	0.298	50.2 (39.3-59.9)	51.8 (40.7-62.1)	<0.001
Male (%)	…	…	…	48.0	46.5	0.1949
Smoking (%)	56.0	3.6	<0.001	29.8	26.1	0.001
Drinking (%)	26.6	2.0	<0.001	14.5	12.3	0.007
Body mass index, kg/m^2^	23.1 (20.9-25.4)	23.1 (21.0-25.6)	0.328	22.4 (20.4-24.6)	24.4 (22.0-27.0)	<0.001
Waist circumference, cm	84.0 (77.0-91.0)	80.0 (74.0-88.0)	<0.001	80.0 (74.0-87.0)	86.0 (79.0-93.0)	<0.001
Systolic blood pressure, mmHg	121.7 (114.0-135.3)	120.0 (110.0-132.7)	<0.001	120.0 (110.0-130.7)	123.3 (116.7-139.3)	<0.001
Diastolic blood pressure, mmHg	80.7 (75.7-89.3)	80.0 (70.7-85.3)	<0.001	80.0 (70.7-86.0)	80.7 (76.7-89.3)	<0.001
Total cholesterol (TC), mmol/l	4.7 (4.2-5.4)	4.8 (4.2-5.6)	<0.001	4.7 (4.1-5.4)	4.9 (4.3-5.6)	<0.001
Triglycerides (TG) , mmol/l	1.3 (0.9-1.9)	1.2 (0.9-1.8)	<0.001	1.1 (0.8-1.6)	1.5 (1.1-2.3)	<0.001
High density lipoprotein cholesterol (HDL-C), mmol/l	1.3 (1.1-1.6)	1.4 (1.2-1.7)	<0.001	1.4 (1.2-1.7)	1.3 (1.1-1.5)	<0.001
Non- HDL-C, mmol/l	3.3 (2.8-4.0)	3.3 (2.7-4.1)	0.936	3.2 (2.6-3.9)	3.6 (3.0-4.3)	<0.001
Low density lipoprotein cholesterol (LDL-C), mmol/l	2.9 (2.3-3.5)	3.0 (2.4-3.6)	<0.001	2.9 (2.3-3.5)	3.0 (2.4-3.7)	<0.001
TC/HDL-C	3.5 (2.9-4.4)	3.3 (2.8-4.1)	<0.001	3.2 (2.7-3.9)	3.8 (3.1-4.6)	<0.001
TG/HDL-C	0.9 (0.6-1.6)	0.8 (0.5-1.3)	<0.001	0.8 (0.5-1.2)	1.2 (0.7-1.9)	<0.001
LDL-C/HDL-C	2.2 (1.6-2.8)	2.1 (1.6-2.6)	<0.001	2.0 (1.5-2.5)	2.3 (1.8-2.9)	<0.001
Non-HDL-C/HDL-C	2.5 (1.9-3.4)	2.3 (1.8-3.1)	<0.001	2.2 (1.7-2.9)	2.8 (2.1-3.6)	<0.001
Apolipoprotein A1, g/l	1.1 (0.9-1.3)	1.1 (1.0-1.3)	<0.001	1.1 (1.0-1.3)	1.1 (0.9-1.3)	<0.001
Apolipoprotein B, g/l	0.9 (0.7-1.1)	0.9 (0.7-1.1)	0.666	0.9 (0.7-1.0)	1.0 (0.8-1.1)	<0.001
Apolipoprotein B/Apolipoprotein A1	0.8 (0.6-1.0)	0.8 (0.6-1.0)	<0.001	0.7 (0.6-0.9)	0.9 (0.7-1.1)	<0.001
Fasting plasma glucose, mmol/l	5.1 (4.7-5.6)	5.0 (4.7-5.5)	0.204	4.9 (4.6-5.3)	5.4 (5.0-6.1)	<0.001
HOMA-IR	2.3 (1.6-3.5)	2.4 (1.6-3.5)	0.091	1.8 (1.3-2.3)	4.2 (3.4-6.2)	<0.001
Uric acid, mmol/l	341.0 (289.0-404.0)	256.0 (213.0-310.0)	<0.001	286.0 (232.0-347.0)	314.0 (255.0-384.0)	<0.001
Hypersensitive C-reactive protein, mg/l	1.0 (1.0-2.0)	1.0 (0.1-2.0)	<0.001	1.0 (0.1-2.0)	1.0 (1.0-3.0)	<0.001

Data are presented as median (interquartile range), or percent.

In both sexes, after controlling for potential intermediate variables, each lipid ratio, and visceral adiposity indicator, as well as TyG considered one-at-a-time made a significant incremental additive contribution to the prediction of HOMA-IR (all P <0.05). The models with lipid ratios were consistently superior to those with the single variables used alone for predicting HOMA-IR (Table 2). For all lipid ratios (TC/HDL-C, TG/HDL-C, LDL-C/HDL-C, non-HDL-C/HDL-C, and apoB/apoA-I), the additional percentage of variation in HOMA-IR explained by each measure ranged from 1 to 2.9% (model 1) in men, and 0.4 to 2.3% (model 1) in women, with log TG/HDL-C being the strongest predictor in both sexes; log apoB/apoA-I was the lipid ratio that had the least association with HOMA-IR and accounted for 1% for men and 0.4% for women (model 1) of the variability in HOMA-IR. For all variables of interest, among both sexes, TyG had the most significant association with HOMA-IR, which explained 9.1% for men and 7.8% for women (model 1) of the variability in HOMA-IR; the variability in HOMA-IR explained by log VAI and log TG/HDL-C was comparable. Results were replicated in models 2 and 3.

**Table 2:** **Multiple linear regression models for predicting homeostatic model assessment**

	**Men**						**Women**					
	**Model 1**		**Model 2**		**Model 3**		**Model 1**		**Model 2**		**Model 3**	
	**Additional R** ^**2**^	**β**	**Additional R** ^**2**^	**β**	**Additional R** ^**2**^	**β**	**Additional R** ^**2**^	**β**	**Additional R** ^**2**^	**β**	**Additional R** ^**2**^	**β**
Lipid measures												
Ln TC	0.001	0.129^*^	0.002	0.168^*^	0.002	0.163^†^	0.001	0.086	0.004	0.229^†^	0.001	0.1
Ln TG	0.027	0.26^†^	0.031	0.239^†^	0.03	0.268^†^	0.022	0.223^†^	0.043	0.286^†^	0.025	0.237^†^
Ln LDL-C	0.001	0.045	0	0.006	0.001	0.066	0.001	0.07^*^	0.002	0.099^*^	0.001	0.071^*^
Ln Non-HD L-C	0.005	0.204^†^	0.007	0.230^†^	0.007	0.243^†^	0.003	0.147^†^	0.011	0.264^†^	0.004	0.166^†^
Ln Apo B	0.003	0.151*	0.004	0.160†	0.004	0.171†	0.006	0.194†	0.014	0.284†	0.006	0.204†
Ln HDL-C	0.009	-0.333^†^	0.013	-0.354^†^	0.014	-0.373^†^	0.005	-0.211^†^	0.01	-0.306^†^	0.006	-0.238
Ln Apo A-I	0.006	-0.235^†^	0.006	-0.237^†^	0.007	-0.261^†^	0	-0.01	0	-0.005	0	-0.014
Lipid ratios												
Ln TC/HDL-C	0.014	0.339^†^	0.017	0.355^†^	0.018	0.383^†^	0.007	0.229^†^	0.019	0.370^†^	0.008	0.257^†^
Ln TG/ HDL-C	0.029	0.212^†^	0.033	0.197^†^	0.033	0.224^†^	0.023	0.170^†^	0.045	0.221^†^	0.026	0.181^†^
Ln LDL-C/HDL-C	0.01	0.160^†^	0.016	0.146^†^	0.015	0.190^†^	0.006	0.157^†^	0.012	0.224^†^	0.007	0.17^†^
Ln Non- HDL-C/HDL-C	0.013	0.227^†^	0.016	0.239^†^	0.017	0.257^†^	0.007	0.163^†^	0.019	0.256^†^	0.009	0.182^†^
Ln Apo B/apo A-I	0.01	0.213^†^	0.011	0.221^†^	0.013	0.237^†^	0.004	0.136^†^	0.01	0.204^†^	0.005	0.145^†^
Visceral adiposity indicators												
Ln LAP	0.017	0.141^†^	0.021	0.143^†^	0.015	0.165^†^	0.016	0.147^†^	0.033	0.195^†^	0.017	0.178^†^
Ln VAI	0.031	0.213^†^	0.034	0.198^†^	0.031	0.217^†^	0.021	0.172^†^	0.042	0.222^†^	0.02	0.173^†^
TyG index	0.091	0.390^†^	0.089	0.347^†^	0.095	0.40^†^	0.078	0.359^†^	0.107	0.381^†^	0.08	0.367^†^

*P <0.05. ^†^P <0.001.

Model 1 was adjusted for age, rural/urban settings, region, education level, smoking status, alcohol use, body mass index, systolic blood pressure, uric acid, and hs-CRP. Model 2 was adjusted for all the variables in model 1 plus waist circumference.

Model 3 was adjusted for body mass index, rural/urban settings, region, education level, alcohol use, and Framingham Risk Score (low, intermediate, and high).

TC, total cholesterol; TG: triglycerides; LDL-C: low density lipoprotein cholesterol; HDL-C: high density lipoprotein cholesterol; Apo B: apolipoprotein B; Apo A-I: apolipoprotein A1; LAP: lipid accumulation product; VAI: visceral adiposity index; TyG index: the product of triglycerides and fasting glucose.

The direct comparative ORs and 95% CIs for those in the top quartiles of each variable were presented in Table 3. For both sexes, after adjustment for age, socioeconomic status, smoking status, alcohol use, BMI, systolic BP, uric acid, and hs-CRP (model 1), TG, apoB, non-HDL-C, HDL-C, each of the lipid ratios, VAI, LAP and TyG were strongly associated with IR (P <0.001), whereas TC, LDL-C, and apoA-I were not associated with IR. Among all lipid parameters, for both sexes, in terms of their strengths of association with IR, lipid ratios performed better overall than any of the individual variables used alone; there was no indication that apoB/apoA-I was superior to traditional lipid ratios; the lipid ratio with the strongest association was TG/HDL-C (top vs bottom OR [95% CI] was 3.04 [2.40-2.85] for men, and 2.70 [2.17-3.36] for women). For all variables of interest, among both sexes, OR for IR with quartile 4 versus quartile 1 was highest for TyG (5.58 for men and 5.18 for women); the effect of VAI, which was superior to LAP, was comparable to TG/HDL-C. Results were largely replicated in models 2 and 3.

**Table 3:** **Adjusted odds ratios of insulin resistance among those in the extreme quartiles of each evaluated variable**

	**Men**			**Women**		
	**Model 1**	**Model 2**	**Model 3**	**Model 1**	**Model 2**	**Model 3**
Lipid measures						
TC	1.23 (0.99-1.52)	1.30 (1.06-1.61)	1.27 (1.03-1.57)	1.17 (0.96-1.44)	1.36 (1.11-1.66)	1.19 (0.97-1.47)
TG	2.78 (2.20-3.51)	2.86 (2.29-3.57)	2.92 (2.31-3.68)	2.70 (2.17-3.36)	3.36 (2.72-4.14)	2.88 (2.32-3.58)
LDL-C	1.13 (0.92-1.39)	1.09 (0.88-1.34)	1.19 (0.96-1.46)	1.13 (0.92-1.39)	1.17 (0.96-1.43)	1.13 (0.92-1.38)
Non-HDL-C	1.64 (1.31-2.05)	1.74 (1.40-2.16)	1.74 (1.40-2.17)	1.46 (1.18-1.81)	1.80 (1.47-2.21)	1.51 (1.22-1.87)
Apo B	1.55 (1.25-1.92)	1.61 (1.30-1.99)	1.59 (1.28-1.97)	1.69 (1.37-2.10)	2.01 (1.63-2.47)	1.69 (1.36-2.09)
HDL-C	0.52 (0.42-0.65)	0.50 (0.41-0.63)	0.49 (0.39-0.61)	0.66 (0.54-0.81)	0.55 (0.46-0.67)	0.64 (0.52-0.78)
Apo A-I	0.70 (0.57-0.86)	0.70 (0.57-0.86)	0.67 (0.54-0.82)	0.93 (0.77-1.13)	0.92 (0.76-1.11)	0.92 (0.76-1.12)
Lipid ratios						
TC/HDL-C	2.14 (1.70-2.68)	2.22 (1.78-2.77)	2.31 (1.84-2.89)	1.87 (1.51-2.32)	2.38 (1.93-2.92)	1.97 (1.59-2.44)
TG/HDL-C	3.04 (2.40-3.85)	3.09 (2.47-3.87)	3.19 (2.53-4.04)	2.70 (2.17-3.36)	3.39 (2.75-4.17)	2.86 (2.30-3.56)
LDL-C/HDL-C	1.57 (1.26-1.95)	1.55 (1.25-1.92)	1.71 (1.38-2.12)	1.57 (1.28-1.93)	1.80 (1.47-2.19)	1.59 (1.29-1.95)
Non- HDL-C/HDL-C	2.14 (1.70-2.68)	2.22 (1.78-2.77)	2.31 (1.84-2.89)	1.87 (1.51-2.32)	2.38 (1.93-2.92)	1.97 (1.59-2.44)
Apo B/apo A-I	1.85 (1.49-2.31)	1.91 (1.54-2.37)	1.96 (1.58-2.43)	1.61 (1.31-1.98)	1.87 (1.53-2.28)	1.63 (1.33-2.01)
Visceral adiposity indicators						
LAP	3.02 (2.39-3.81)	3.06 (2.45-3.82)	3.21 (2.52-4.08)	2.31 (1.79-2.98)	3.01 (2.38-3.81)	2.59 (2.07-3.23)
VAI	3.1 (2.35-4.07)	3.16 (2.43-4.18)	3.76 (2.7-5.24)	2.45 (1.98-3.05)	3.12 (2.54-3.82)	2.86 (2.13-3.84)
TyG index	5.58 (4.37-7.14)	5.29 (4.19-6.69)	5.74 (4.49-7.33)	5.18 (4.10-6.54)	5.91 (4.75-7.35)	5.37 (4.25-6.77)

Model 1 was adjusted for age, rural/urban settings, region, education level, smoking status, alcohol use, body mass index, systolic blood pressure, uric acid, and hs-CRP. Model 2 was adjusted for all the variables in model 1 plus waist circumference.

Model 3 was adjusted for body mass index, rural/urban settings, region, education level, alcohol use, and Framingham Risk Score (low, intermediate, and high).

TC, total cholesterol; TG: triglycerides; LDL-C: low density lipoprotein cholesterol; HDL-C: high density lipoprotein cholesterol; Apo B: apolipoprotein B; Apo A-I: apolipoprotein A1; LAP: lipid accumulation product; VAI: visceral adiposity index; TyG index: the product of triglycerides and fasting glucose.

Areas under the ROC curves derived from lipid ratios were in general significantly greater than from single lipids (Table 4). For all lipid ratios, the AUC for the TG/HDL-C was greatest in both sexes (0.665 in men and 0.664 in women). For comparison, the AUC for apoB/apoA-I was 0.625 in men and 0.613 in women. For all variables of interest, among both genders, TyG presented the greatest value of AUC (0.709 in men and 0.711 in women); the AUC for VAI (0.695 in men and 0.682 in women) was greater than that for TG/HDL-C.

**Table 4:** **Areas under the receiver operating characteristic (ROC) curves for each evaluated variable in predicting homeostatic model assessment**

	**Men**	**Women**
Lipid measures		
TC	0.558 (0.539-0.578)	0.566 (0.547-0.584)
TG	0.657 (0.638-0.675)	0.662 (0.645-0.679)
LDL-C	0.538 (0.519-0.558)	0.550 (0.532-0.569)
Non-HDL-C	0.606 (0.587-0.625)	0.602 (0.584-0.619)
Apo B	0.596 (0.577-0.615)	0.608 (0.590-0.627)
HDL-C	0.376 (0.357-0.395)	0.402 (0.384-0.420)
Apo A-I	0.427 (0.408-0.446)	0.471 (0.453-0.490)
Lipid ratios		
TC/HDL-C	0.568 (0.549-0.578)	0.576 (0.557-0.584)
TG/HDL-C	0.665 (0.647-0.684)	0.664 (0.647-0.681)
LDL-C/HDL-C	0.611 (0.592-0.630)	0.606 (0.588-0.624)
Non- HDL-C/HDL-C	0.646 (0.627-0.664)	0.632 (0.615-0.650)
Apo B/apo A-I	0.625 (0.606-0.644)	0.613 (0.595-0.631)
Visceral adiposity indicators		
LAP	0.671 (0.652-0.689)	0.666 (0.649-0.684)
VAI	0.695 (0.677-0.713)	0.682 (0.665-0.699)
TyG	0.709 (0.692-0.726)	0.711 (0.695-0.728)

TC, total cholesterol; TG: triglycerides; LDL-C: low density lipoprotein cholesterol; HDL-C: high density lipoprotein cholesterol; Apo B: apolipoprotein B; Apo A-I: apolipoprotein A1; LAP: lipid accumulation product; VAI: visceral adiposity index; TyG index: the product of triglycerides and fasting glucose.

Since WC has a strong effect on IR and was sensitive for discriminating lipids abnormalities [21], we selected the 3 variables with the greatest AUCs (TG/HDL-C, VAI, and TyG), which also explained the most variability in HOMA-IR and had the highest ORs in the fourth quartile, to investigate whether the relationships between each of the 3 variables and IR differed by WC status (central obese and non-central obese). After adjusting for BMI, socioeconomic status, alcohol use, and FRS, all the 3 variables remained significant predictors of IR irrespective of WC status (Table 5). Moreover, the interactions between WC levels and each of the 3 variables on IR did not reach statistical significance, indicating that the associations of each of the 3 variables with IR did not differ by WC status.

**Table 5:** **Odds ratios for homeostatic model assessment according to levels of waist circumference and TG/HDL, VAI, or TyG**

		**Men**				
		**Q1**	**Q2**	**Q3**	**Q4**	**P** ^*^
TG/HDL	WC <90 cm	1	1.43 (1.11-1.84)	1.81 (1.40-2.35)	3.23 (2.47-4.22)	0.779
	WC ≥90 cm	1.29 (0.84-1.97)	1.86 (1.29-2.68)	2.65 (1.90-3.70)	3.73 (2.73-5.11)	
VAI	WC <90 cm	1	1.30 (1.02-1.67)	1.69 (1.31-2.18)	3.13 (2.40-4.08)	0.943
	WC ≥90 cm	1.10 (0.70-1.75)	1.60 (1.11-2.30)	2.39 (1.73-3.29)	3.35 (2.48-4.53)	
TyG	WC < 90 cm	1	1.65 (1.26-2.17)	2.60 (1.99-3.39)	5.60 (4.24-7.40)	0.408
	WC ≥90 cm	1.22 (0.77-1.92)	2.41 (1.66-3.48)	3.54 (2.51-4.97)	5.95 (4.30-8.21)	
		**Women**				
TG/HDL	WC <80 cm	1	1.43 (1.07-1.90)	1.70 (1.26-2.29)	3.97 (2.89-5.47)	0.793
	WC ≥80 cm	1.23 (0.89-1.69)	1.59 (1.18-2.14)	2.57 (1.93-3.41)	3.82 (2.88-5.08)	
VAI	WC <80 cm	1	1.25 (0.95-1.65)	1.55 (1.16-2.08)	3.85 (2.79-5.31)	0.864
	WC ≥80 cm	1.13 (0.82-1.55)	1.29 (0.97-1.72)	2.23 (1.71-2.91)	3.15 (2.42-4.11)	
TyG	WC < 80 cm	1	1.55 (1.15-2.09)	2.46 (1.81-3.34)	6.72 (4.83-9.34)	0.634
	WC ≥80 cm	1.08 (0.76-1.54)	1.94 (1.43-2.64)	3.18 (2.38-4.26)	6.09 (4.54-8.18)	

^*^P for interaction terms (TG/HDL × WC status, VAI × WC status, and TyG × WC status) were assessed by logistic regression analysis.

TG: triglycerides; WC, waist circumference; HDL-C: high density lipoprotein cholesterol; VAI: visceral adiposity index; TyG index: the product of triglycerides and fasting glucose.

Results were remarkably similar when IR was defined by HOMA2-IR as greater than the 75th percentile, which was calculated using the HOMA2 calculator updated in 2007 (data not shown).

## Discussion

In the present study, we directly compared lipid and apolipoprotein measures, lipid ratios, LAP, VAI, and TyG as predictors of IR. Overall, we observed that lipid ratios were superior to either pro-atherogenic or anti-atherogenic fractions alone. The apoB/apoA-I performed no better than any of the traditional lipid ratios. Of the lipid ratios, the magnitude of the association was greatest for TG/HDL-C. TyG appears to be most closely associated with IR among all the variables studied. VAI and TG/HDL-C were equivalent in their relationships with IR. Moreover, ROC curve analysis confirmed these observations by indicating that TG/HDL-C was better than apoB/apoA-I in discriminating IR and TyG was the best discriminator in identifying IR.

There have been several reports suggest that traditional lipid ratios have greater predictive power for IR and CVD risk than each of the single standard lipid measures [2,22,23]. However, there has been very few study address the crucial question of whether apoB/apoA-I predicts IR better than traditional lipid ratios do. Plasma apoB concentration provides a measure of the concentration of all potentially atherogenic particles (Each very low–density lipoprotein, intermediate-density lipoprotein, and LDL particle is covered by 1 apoB molecule), and increased small dense LDL particles is related with IR and CVD risk. On the other hand, apoA-I is the main structural protein of HDL particles. Consequently, apoB/apoA-I has been recommended as an effective indicator to assess CVD risk [7]. However, the question of whether apoB/apoA-I should be introduced into guidelines as a target for therapy in people with dyslipidemia is a debated issue, as some reports indicated that apoB/apoA-I were better predictors of CVD risk compared with traditional lipid ratios [22,23], and other studies reported that apoB/apoA-I and non-HDL-C/HDL-C were comparable in terms of predicting CVD risk [8,24]. Accumulating evidence indicates that IR facilitates the development of CVD. However, data on direct comparisons between traditional lipid ratios and apoB/apoA-I to identify IR is limited. Despite the evidence that apoB/apoAI ratio is significantly associated with IR [6], we found apoB/apoA1apoA-I has little to offer over traditional lipid ratios (TC/HDL-C, LDL-C/HDL-C, and non-HDL-C/HDL-C) in predicting IR. One possible explanation for this result may be that 90% of apoB-carrying particles are found on LDL, irrespective of their size. It has been indicated that LDL-C has little correlation with IR. Moreover, the OR for TG/HDL-C was greater than that for apoB/apoA-I. Furthermore, evidence shows that two simple algorithms to calculate apoB from routine lipids are effective in estimating measured apoB [25].Taken together, these results provide little support for replacing measurement of traditional lipid ratios with measurement of the apoB/apoA-I ratio as risk prediction tools. This information is of pragmatic interest, as traditional lipid ratios can be directly calculated from traditional lipid measures at no incremental cost.

Although lipoproteins and apolipoproteins were not measured by the more sophisticated method nuclear magnetic resonance spectroscopy, emerging evidence suggested that the association of coronary artery calcification with nuclear magnetic resonance-measured lipoproteins was comparable to that with standard lipids [26]. It has been reported that the sensitivity and specificity for TG/HDL-C in identifying IR are virtually identical to those for fasting insulin concentration [27]. Furthermore, TG/HDL-C has been shown to have a stronger correlation with IR when compared with other traditional lipid or lipid ratios [28,29]. However, correlations between TG/HDL-C and IR differ according to ethnic origin [30]. Thus, it is possible that how well TG/HDL-C correlates with IR depends on the target population. Our study appears to be the first Chinese population-based study using a large database of cohort for exploring whether TG/HDL-C is a better correlate of IR compared with other lipid measures. Consistent with other studies [28,29,31], we found TG/HDL-C was a better marker for IR than any of other lipid parameters. Given that standard assay for TG and HDL-C are widely available in clinical practice, and that it is simple to calculate TG/HDL-C from TG and HDL-C, as well as that TG/HDL-C is superior to other lipid parameters in predicting IR, it is reasonable to recommend TG/HDL-C as an effective and convenient indicator of IR.

Since the concurrence of hypertriglyceridemia and low HDL-C are key metabolic abnormalities in patients with IR and represent diabetic dyslipidemia, it is expected that we found a superiority of TG/HDL-C over other lipid measures in correlating IR. However, it is worth emphasizing that variations in several parameters (levels of lecithin:cholesterol acyltransferase, post-heparin hepatic lipase activities, post-heparin lipoprotein lipase activities, phospholipid transfer protein) that control the plasma lipoprotein metabolism are related to an increased degree of insulin sensitivity among healthy (BMI <30 Kg/m^2^) high HDL-C (>60 mg/dL) subjects [32]. Binding and internalization rates of TG-enriched HDL in these subjects may slow down [33], inducing a relatively lower level of IR.

Our study is the only one directly comparing the utility of VAI with different lipid measures in assessing IR risk. We found that the VAI, which take account of the concentrations of TG and HDL-C, as well as adiposity measures (BMI and WC), and TG/HDL-C have comparable associations with IR. However, when we used ROC analysis to determine the ability of these 2 indices to discriminate between persons with and without IR, we found that VAI was a better discriminator, indicating that VAI offers more clinical information than that achieved by using TG/HDL-C. The better ability of VAI to identify IR individuals compared with TG/HDL-C is of clinical relevance and can be possibly explained by the fact that the VAI takes regional body fat distribution into consideration. VAI is a good indicator of visceral adiposity [11] and visceral fat is more metabolically deleterious than subcutaneous fat [13,34]. According to the overflow hypothesis, the subcutaneous fat depot appears to act as a protective metabolic sink, storing dietary fat to limit their deposition in undesired sites such as liver, heart, skeletal muscle, and pancreatic β cells [34]. However, for some individuals with more visceral fat and less subcutaneous fat, available lipids exceed the subcutaneous adipose tissue’s capacity for buffering and storage, the excess of lipids will be reoriented toward nonadipose tissues [35]. The ectopic lipid deposits lead to lipotoxicities, incurring insulin resistance. Hence, obesity is a remarkably heterogeneous disorder, as evidenced by the occurrence of a subset of obese persons who are insulin sensitive, and a subset of normal-weight people who are insulin resistant [36]. It is with the heterogeneity in mind that measuring an indicator of visceral fat such as VAI is clinically benefit, as visceral obesity is more important in modulating IR [9,13]. In the current study, LAP, another indicator of visceral adiposity, does not offer any clinical benefit as compared with TG/HDL-C. Although explanations for this issue remain to be elucidated, it is probably related to the fact that LAP does not incorporate anti-atherogenic lipoproteins.

TyG has been recently recommended as a simple and inexpensive index to evaluate IR [14,15]. Because of the variability of TG levels according to ethnicity, it is necessary to assess the utility of TyG in predicting IR in a Chinese population. The current analysis was initiated to perform the task. We found that the TyG index was a good discriminator in predicting IR and was a better correlate of IR than TG/HDL-C, a finding consistent with results from other studies [14,15]. Moreover, our study extends previous studies by directly comparing the utility of TyG with visceral adiposity indicators and other lipid parameters in assessing IR risk. We found that TyG was the best index in discriminating IR individuals, highlighting that TyG can serve as a simple index for identifying individuals with a high risk of IR. The superiority of TyG to any of the other indices we studied can be possibly explained by the fact that glucotoxicity and lipotoxicities are key mechanisms in modulation of IR. A recent study indicated that 20/(fasting C-peptide immunoreactivity × fasting glucose) outperformed HOMA-IR for the assessment of glucose infusion rate measured with the hyperinsulinemic-euglycemic glucose clamp [37]. Since fasting C-peptide immunoreactivity was not available in CHNS 2009, we have no way to validate the usefulness of TyG in identifying IR evaluated with 20/(fasting C-peptide immunoreactivity × FPG), and further research with data of fasting C-peptide immunoreactivity is warranted.

Our study has several strengths including a well-established cohort of Chinese population, a vigorous quality assurance program and the same strict methodology used to ensure the quality of the data collection over the entire study period. In addition, we evaluated the performance of traditional lipid ratios, apo B/apo A-I ratio, VAI, LAP, and TyG in correlating with IR across the full glycemic spectrum (from normal glucose tolerance to impaired glucose tolerance to diabetes). Nevertheless, the present study has several limitations. The study population is comprised of only Chinese adults, thus, extrapolating results to other racial or ethnic population should be interpreted cautiously. The cross-sectional design implies that no potential temporal relations can be drawn. Hyperinsulinemic-euglycemic clamp, the gold standard for evaluating insulin sensitivity, was not conducted. However, HOMA-IR, a surrogate of IR, has been shown to correlate well with IR index derived from the euglycemic clamp [38]. The fact that we have no way to evaluate the associations of TG/HDL-C and apoB/apoA-I ratio with IR according to LDL subfraction phenotype is another limitation, as phenotype B is associated with increased oxidized LDL, glycated LDL, and lipoprotein-associated phospholipase A2 (also known as platelet-activating factor acetylhydrolase) activity [39] and with increased CVD risk [40].

## Conclusions

Apolipoprotein ratios performed no better than any traditional lipid ratio in predicting IR risk. The TG/HDL-C ratio, VAI and TyG index are effective discriminators in predicting IR. This information is of particular clinical relevance, as all these 3 indices can be calculated from common measures such as TG, FPG, and HDL-C, which are based on standardized measurements and widely available. The ability of TG/HDL-C to identify IR seems compromised by taking account of body fat distribution. The TyG index is the best marker predicting the risk of IR.

## Abbreviations

TC: Total cholesterol
HDL-C: High density lipoprotein cholesterol
TG: Triglycerides
ApoB: Apolipoprotein B
ApoA-I: Apolipoprotein A-I
VAI: Visceral adiposity index
LAP: Lipid accumulation product
TyG: The product of TG and fasting glucose
IR: Insulin resistance
ROC curve: Receiver operating characteristic curve
AUC: Area under the ROC curve
BMI: Body mass index
WC: Waist circumference
CHNS: China Health and Nutrition Survey
BP: Blood pressure
WHO: World Health Organization
SD: Standard deviation
